# EEG Microstate Differences Between Alzheimer’s Disease, Frontotemporal Dementia, and Healthy Controls Using 4 and 7 Clustering Classes with a Ratio Approach

**DOI:** 10.3390/medicina61111917

**Published:** 2025-10-25

**Authors:** Jinwon Chang

**Affiliations:** 1 Department of Psychology, Williams College, 39 Chapin Hall Drive, Williamstown, MA 01267, USA; jc49@williams.edu; Tel.: +1-(857)-268-3527; 2 Department of Psychiatry, Beth Israel Deaconess Medical Center, Harvard Medical School, Boston, MA 02215, USA

**Keywords:** Electroencephalography, Alzheimer’s disease, frontotemporal dementia, microstates

## Abstract

*Background and Objectives*: Alzheimer’s disease (AD) and frontotemporal dementia (FTD) present overlapping clinical and neuroanatomical features, complicating early diagnosis. Therefore, this study evaluated whether EEG microstate analysis can provide reliable markers to distinguish patients with dementia from healthy controls. *Materials and Methods*: Resting-state EEG was recorded from 36 AD patients, 23 FTD patients, and 29 healthy controls. Preprocessing and microstate analysis were conducted using the MICROSTATELAB pipeline in EEGLAB. Clustering solutions ranging from four to seven classes were tested, with grand mean fitting and variance thresholds. Temporal parameters (duration, occurrence, and coverage) and their ratio-normalized forms were compared across groups using ANCOVA and nonparametric tests. Associations with Mini-Mental State Examination (MMSE) scores were assessed by regression analyses. *Results*: The four- and seven-class clustering solutions achieved high variance overlap with published microstate templates. In the four-class solution, temporal parameters of microstates B and D significantly differentiated controls from dementia groups, while in the seven-class solution, microstates C and G were the most informative. Ratio-normalized parameters improved group discrimination and were associated with MMSE scores. *Conclusions*: EEG microstates capture disease-related alterations in large-scale brain dynamics that differentiate patients with dementia from healthy individuals.

## 1. Introduction

Alzheimer’s disease (AD) and frontotemporal dementia (FTD) are among the most common forms of dementia, both profoundly impairing cognitive and behavioral abilities. AD typically affects both amnestic (learning and memory) and non-amnestic domains (executive function and visuospatial abilities), whereas FTD more often disrupts interpersonal conduct and self-regulation [1,2]. Clinically, however, differentiating AD from FTD remains challenging. Both disorders show overlapping patterns of neurodegeneration in the dorsolateral prefrontal cortex and medial temporal lobes, including the amygdala and hippocampus, and behavioral symptoms may overlap, particularly between the behavioral variant of FTD (bvFTD) or primary progressive aphasia and atypical AD [3,4]. Although MRI and biomarker assays such as cerebrospinal fluid (CSF) and positron emission tomography (PET) can aid diagnosis, these methods are costly, invasive, and limited in terms of temporal resolution and early detection abilities.

Electroencephalography (EEG) offers a cost-effective, noninvasive, and widely accessible alternative for assessing brain function. Conventional EEG analyses typically focus on spectral power or functional connectivity, but these approaches have limitations. Functional connectivity shows poor test–retest reliability [5], while spectral analyses are confounded by baseline correction issues and broadband activity [6].

In recent years, EEG microstate analysis has emerged as a promising approach to characterizing large-scale brain dynamics during rest [7]. Microstates are quasi-stable scalp potential configurations lasting tens of milliseconds before rapidly transitioning, thought to reflect the sequential activation of large-scale neural networks [8]. Canonical microstates (A–D) are associated with distinct topographies, temporal features, and putative cognitive functions (e.g., microstate A is linked to phonological processing, microstate B to the visual network, microstate C to the salience network, and microstate D to attentional networks) [9]. Importantly, microstate parameters such as duration, occurrence, and coverage demonstrate high test–retest reliability across sessions, montages, and clustering procedures, underscoring their potential as biomarkers [10].

Several studies have examined EEG microstates in AD and FTD. For example, one study reported a shorter microstate C duration in FTD compared with that in AD and controls [11]. Another study observed an increased microstate B and microstate A duration in early-onset AD and FTD, respectively, along with reduced occurrences of microstates A–C in early-onset AD and reduced microstate C occurrence in FTD [12]. Also, another study found that patients with semantic dementia lacked a typical microstate C but exhibited an atypical microstate E, which might indicate dysfunctional cognitive function integration [13].

Despite these findings, methodological shortcomings limit their interpretation. For example, many prior studies did not apply grand mean fitting or achieved less than 80% explained variance [12], while others lacked group mean maps or grand mean templates [11,13]. The lack of using global maps in these microstate analyses reduces their reliability and comparability because individual template maps introduce spatial variance across subjects and groups, which eventually increases the variance of microstates’ extracted characteristics [14]. Moreover, most studies constrained analyses to four canonical microstates a priori, though recent work suggests that optimal clustering often exceeds four states, extending to 4–7 maps to better represent complicated networks of brain dynamics through more frequently alternating microstate maps with different topographies [10,15].

Together, these inconsistencies highlight the need for standardized analysis pipelines. To address this gap, in the present study, we apply a validated microstate workflow in MICROSTATELAB in EEGLAB [16], focusing only on temporal parameters demonstrated to be reliable [10]. Additionally, we introduce a ratio approach that normalizes each microstate parameter to the total across all microstates (e.g., relative duration of microstate A = duration of A ÷ total duration). This adjustment reduces inter-individual variability such as individual background activity (also known as 1/f noise) [6] and may improve sensitivity for detecting group differences.

## 2. Materials and Methods

### 2.1. Participants

The study included 36 patients with AD, 23 with FTD, and 29 healthy controls (CTL) [17]. The original dataset can be found in the publicly available OpenNeuro database: https://doi.org/10.18112/openneuro.ds004504.v1.0.8. Cognitive status was assessed using the Mini-Mental State Examination (MMSE) [18], which ranges from 0 to 30, with lower scores indicating more severe decline. The median disease duration was 25 months (IQR: 24–28.5), representing the early or mild stages of dementia. No dementia-related comorbidities were reported in the AD or FTD group. Diagnoses were made according to the Diagnostic and Statistical Manual of Mental Disorders, 3rd ed., revised (DSM-IIIR, DSM IV, ICD-10) [19] and the National Institute of Neurological, Communicative Disorders and Stroke—Alzheimer’s Disease and Related Disorders Association (NINCDS—ADRDA) [20]. Mean MMSE scores were 17.75 (SD = 4.5) in the AD group, 22.17 (SD = 8.22) in the FTD group, and 30 in controls. Mean ages were 66.4 years (SD = 7.9; min = 49; max = 79) for AD, 63.6 (SD = 8.2; min = 44; max = 78) for FTD, and 67.9 (SD = 5.4; min = 57; max = 78) for controls. Individual information on these clinical demographical characteristics can be found in the publicly available dataset.

### 2.2. EEG Acquisition and Preprocessing

Resting-state EEG was recorded at the 2nd Department of Neurology, AHEPA General Hospital, Thessaloniki, by an experienced neurology team. Signals were acquired with a Nihon Kohden 2100 system using 19 scalp electrodes (Fp1, Fp2, F7, F3, Fz, F4, F8, T3, C3, Cz, C4, T4, T5, P3, Pz, P4, T6, O1, O2) placed according to the 10–20 system. Mastoids (A1, A2) were used as references and for impedance checks. Participants sat with their eyes closed during recordings. Data were sampled at 500 Hz with 10 μV/mm resolution and recordings were referenced to Cz for common-mode rejection. The study was approved by the Scientific and Ethics Committee of AHEPA University Hospital, Aristotle University of Thessaloniki (Protocol No. 142/12 April 2023) and conducted in accordance with the Declaration of Helsinki.

Preprocessing was performed in EEGLAB (version 2025.0.0) [21], in which signals were band-pass-filtered (2–20 Hz) with an FIR filter following MICROSTATELAB recommendations [15] and reliability findings [10]. Artifact Subspace Reconstruction (ASR) was applied with a 0.5 s sliding window and 20 SD threshold. Bad segments were removed using RMS thresholds with a 25% channel-outlier criterion. Data were re-referenced to the average; then, independent component analysis (ICA) was performed, and components with ≥70% probability of ocular or muscle origin were rejected.

### 2.3. Microstate Analysis

Microstate analysis was conducted using MICROSTATELAB [15]. Individual templates were identified with a k-means clustering algorithm with 20 restarts, applied to global field power (GFP) peaks while ignoring polarity. Clustering and backfitting microstates using GFP were performed by selecting only maps with momentary GFP peaks for the optimal signal-to-noise ratio while assuming that oscillatory sources and constant background noise compose all relevant signals [15]. Remaining data are labeled according to the nearest GFP label. Clustering was performed for solutions ranging from four to seven classes. Group mean maps were then computed, followed by a grand mean map across all participants, which were sorted according to the spatial correlation across maps by optimizing the sequence for maximum shared variance with the previously established EEG microstate metamap [14]. The optimal number of classes was defined as the solution yielding more than 80% shared variance for each microstate map with established templates; both the four- and seven-class solutions met this criterion and were therefore retained. Grand mean maps were used to facilitate sorting of all dependent maps. GFP peaks and grand mean templates were applied for backfitting to compute temporal parameters. Duration was defined as the mean length of each microstate, occurrence as the average number per second, and coverage as the percentage of total EEG time occupied by each microstate. Transition probabilities were not analyzed because of their poor test–retest reliability [10]. The same processing procedures were applied for both the four- and seven-class clustering methods. Relative duration and occurrence were also calculated by dividing the duration or occurrence of each microstate by the total across all microstates post hoc (e.g., (relative duration of microstate A) = (duration of microstate A)/(duration of all microstates)).

### 2.4. Statistical Analysis

Statistical analyses were performed in MedCalc (version 23.0.8; MedCalc Software Ltd., Ostend, Belgium), MATLAB (version R2023a), and JASP (version 0.19.3). Between-group differences were tested using ANCOVA with age and gender as covariates and with the Kruskal–Wallis test followed by Dunn’s post hoc comparisons. Bonferroni correction was applied for multiple testing for all microstates within each clustering solution. Associations between MMSE scores and microstate parameters were examined using linear regression with age and gender included as covariates.

## 3. Results

**Figure 1:**
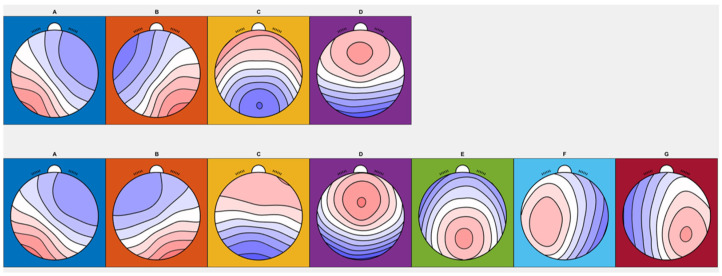
EEG grand mean microstate maps of subjects. For four-class clustering solutions, microstates (**A**–**D**) were identified in the first line, while microstates (**A**–**G**) were identified for seven-class clustering solutions in the second line.

Across the participants, both the four- and seven-class clustering solutions were identified, and the resulting maps were consistent with those already published [10] (Figure 1). For the four-class solution, the shared variance with the published metamaps was 97.35%, 98.70%, 96.86%, and 86.54% for microstates A, B, C, and D, respectively (Table 1).

The group comparisons revealed significant differences in the temporal parameters of several microstates. In the four-class solution, the ANCOVA and Kruskal–Wallis analyses indicated that the duration, coverage, relative duration, and relative occurrence of microstate B, as well as the occurrence and relative duration of microstate D, differed significantly across the CTL, FTD, and AD groups (*p* < 0.05 for both parametric and nonparametric comparisons) (Table 2).

**Figure 2:**
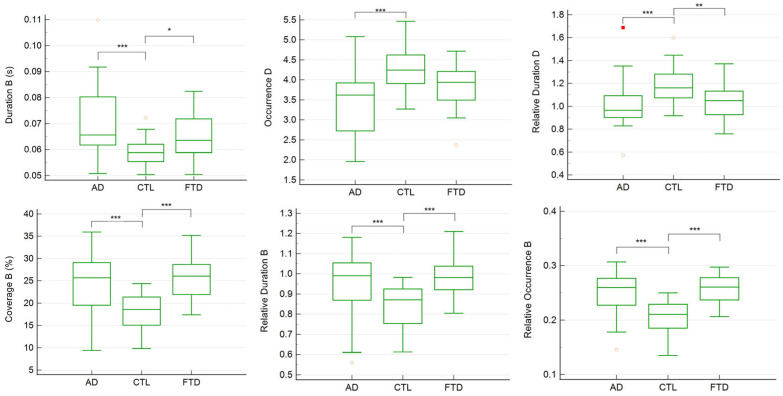
Individual comparisons of each significant temporal parameter across CTL, FTD, and AD groups in 4-class microstates. In the box-and-whisker plot, yellow hollow dots represent outside values (smaller than lower quartile minus 1.5 times the interquartile range or larger than the upper quartile plus 1.5 times the interquartile range (inner fences)). Red rectangles represent far-out values (smaller than the lower quartile minus 3 times the interquartile range, or larger than the upper quartile plus 3 times the interquartile range (outer fences)). The three asterisks represent *p* value < 0.001; two asterisks represent *p* value < 0.01; one asterisk represents *p* value < 0.05.

The CTL group showed a significantly lower duration, coverage, relative duration, and relative occurrence in microstate B compared with both the AD and FTD groups (*p* < 0.001, except for the duration of microstate B, where CTL vs. FTD showed *p* < 0.05). The occurrence of microstate D was significantly higher in the CTL group compared to the AD group (*p* < 0.001) but not the FTD group, while the relative duration of microstate D was significantly higher in the CTL group compared to both the AD and FTD groups (*p* < 0.001 and *p* < 0.01, respectively) (Figure 2).

Linear regression analyses controlling age and gender confirmed that these temporal parameters were strongly related to the MMSE scores (Table 3). Specifically, the duration, coverage, relative duration, and relative occurrence of microstate B, as well as the occurrence and relative duration of microstate D, were significant predictors of MMSE performance.

For the seven-class solution, the shared variance with published metamaps was 99.06%, 87.99%, 95.63%, 95.85%, 92.74%, 97.81%, and 92.17% for microstates A through G, respectively (Table 4). The ANCOVA and Kruskal–Wallis analyses identified significant group differences in the duration, coverage, relative duration, and relative occurrence of microstate G and the occurrence, coverage, relative duration and relative occurrence of microstate C (*p* < 0.05 for both parametric and nonparametric comparisons) (Table 5).

**Figure 3:**
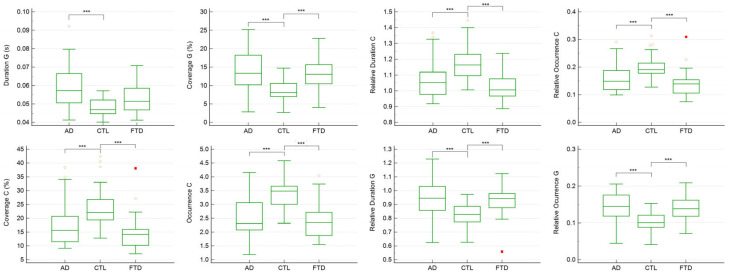
Individual comparisons of each significant temporal parameter across CTL, FTD, and AD groups in 7-class microstates. In the box-and-whisker plot, yellow hollow dots represent outside values (smaller than lower quartile minus 1.5 times the interquartile range or larger than the upper quartile plus 1.5 times the interquartile range (inner fences)). Red rectangles represent far-out values (smaller than the lower quartile minus 3 times the interquartile range, or larger than the upper quartile plus 3 times the interquartile range (outer fences)). Three asterisks represent *p* value <0.001.

In particular, the duration of microstate G was significantly longer in the AD group compared to that in the CTL group (*p* < 0.001). Moreover, the coverage, occurrence, relative duration, and relative occurrence of microstate C were significantly higher in the CTL group compared to that in the AD and FTD groups, while the coverage, relative duration, and relative occurrence of microstate G were all significantly lower in the CTL group compared to the AD and FTD groups (*p* < 0.001) (Figure 3).

The regression analyses further demonstrated that the duration, coverage, relative duration, and relative occurrence of microstate G, together with the occurrence, coverage, relative duration, and relative occurrence of microstate C, were significantly associated with the MMSE scores (Table 6).

## 4. Discussion

The present study demonstrates that different clustering solutions in EEG microstate analysis reveal distinct but complementary markers of dementia. Using four-class clustering, the temporal parameters of microstates B and D differentiated healthy controls from both the AD and FTD groups. In contrast, with seven-class clustering, the temporal parameters of microstates C and G distinguished the controls from the dementia groups. In both clustering solutions, these microstate parameters were also significantly associated with cognitive status, as reflected in the MMSE scores, indicating that microstate parameters are associated with cognitive impairments in patients with dementia. Importantly, applying relative measures of duration and occurrence provided additional discriminatory power. For example, in the seven-class solution, the absolute duration of microstate C and the occurrence of microstate G did not significantly differ between groups, whereas their relative values successfully distinguished healthy controls from patients with dementia.

Overall, the results of the current study are not fully consistent with those reported in the previous literature. Unlike one previous study that discovered a shorter microstate C duration in patients with FTD [11], there was no significance difference between the groups in terms of this parameter in our study. Also, in another study, increased microstate A duration and decreased microstate A occurrence were observed in the FTD and AD groups, respectively, but no difference in microstate A was discovered in the current study [12]. However, some consistent findings include reduced occurrences of microstate C in early-onset AD and FTD [12]. Such differences between studies might be derived from the use of different analysis procedures and parameters, which supports the importance of a standardized procedure, as was applied in the current study.

Although four- and seven-class solutions capture different numbers and topographies of microstates, both sets of maps showed a high shared variance with published template maps from meta-analyses [14]. This correspondence supports the validity of our clustering results and aligns with the broader view that microstates reflect brief but functionally meaningful configurations of large-scale brain networks. Functional MRI studies have suggested that canonical microstates correspond to resting-state networks (RSNs), with microstate A linked to phonological processing, microstate B to the visual network, microstate C to the salience network, and microstate D to attentional networks [22,23]. Also, in the extended microstate maps, microstate E represents the interoceptive and emotion processing network, partially overlapping with the salience network in microstate C; microstate F reveals areas of the anterior default mode network that is responsible for the theory of mind and information processing; and microstate G is related to the somatosensory network [24]. According to this framework, spontaneous brain activity alternates rapidly across RSNs, and microstates capture these transitions on the millisecond scale [25]. From this perspective, the “duration” of the microstate reveals the stability of each RSN, the “occurrence” represents the frequency of activation of its RSNs, and the “coverage” indicates the relative time coverage of the related RSNs [9]. However, mapping between microstates and RSNs remains controversial since EEG microstates operate on much shorter time scales than fMRI. Some studies report an association between microstates and the default mode network [26], while others have not confirmed this relationship [22]. Consequently, microstates that appear topographically similar across different clustering solutions, such as those labeled “B,” may nonetheless index distinct underlying RSNs. Therefore, the same label in the four- and seven-class clustering solutions in the current study might represent different functional correlates, which needs further investigation with comparisons to different neuroimaging techniques. This indicates that considering multiple clustering solutions may be important for fully characterizing the range of network dynamics in EEG microstate analysis.

Another novel strength of the present study is the use of ratio-normalized parameters, in which the duration or occurrence of each microstate is expressed relative to the total across all microstates. This approach reduces inter-individual variability, which is known to affect EEG microstate measures [26], and may provide a more reliable index of temporal dynamics. Each temporal parameter captures a different aspect of large-scale network activity: duration reflects microstate stability, occurrence reflects the switching rate, and coverage reflects the relative dominance of a microstate. By normalizing duration and occurrence, we may more effectively capture relative changes in network stability and switching that accompany dementia. Although further work is needed to establish the precise neurophysiological meaning of relative parameters, our findings suggest that they provide useful complementary information for distinguishing clinical groups.

Our results further indicate that EEG microstates can serve as early and cost-effective screening markers for dementia. While they did not differentiate between AD and FTD, the microstate parameters we identified reliably distinguished both patient groups from healthy controls. The lack of a differential diagnosis between AD and FTD might be due to the overlapping patterns of neurodegeneration and behavioral symptoms [3,4]. As each subtype of AD and FTD represents different network dysfunctions and related biomarkers [1,2], further study is needed to investigate the effectiveness of EEG microstate parameters in classifying these dementia subtypes. For example, EEG microstate analysis of bvFTD might show abnormalities in microstate D due to its dysfunction in personality and behavior, while that of the semantic variant of FTD (primary progressive aphasia) might demonstrate a difference in microstate A, which is often related to language processing [22,23]. Nevertheless, our result aligns with recent recommendations highlighting the potential of EEG biomarkers for dementia diagnosis [27]. A stratified diagnostic approach could therefore be envisioned, in which EEG microstate analysis is used as an inexpensive and noninvasive first-line screening tool to identify individuals at risk, followed by more costly or invasive methods such as MRI or PET for diagnostic confirmation. For example, patients with probable dementia could be initially screened with convenient EEG microstate analysis, and then more complex neuroimaging methods could be applied to identify the exact type of dementia. Such a tiered approach would lower costs, improve accessibility, and facilitate earlier detection of this condition.

There are some limitations to our study, however. The small sample size and the retrospective design employed complicate the practical evaluation of microstate parameters as diagnostic biomarkers. Further investigations with larger sample sizes, prospective designs, and multi-center cohorts with more detailed clinical information such as subtypes of dementia are needed to fully evaluate whether microstate parameters are effective in classifying neurological abnormalities. Also, there is no patient information on biomarkers such as amyloid and tau in cerebrospinal fluid or PET included in the current study. This limits further comparison with other biomarkers and provides a pathophysiological perspective on patients with dementia related to EEG microstate parameters. Additionally, provided that MMSE scores are associated with our significant parameters, further investigation is needed to elucidate the relationship between our significant parameters and other clinical scores to discover their cognitive correlates.

Nevertheless, the current study is valuable as an exploratory analysis of EEG microstates as promising dementia biomarkers. EEG has been investigated for decades as a useful source of biomarkers of neurological disorders. For example, the spectral theta/beta ratio has been approved by the Food and Drug Administration (FDA) for the assessment of attention-deficit hyperactivity disorder (ADHD) as a supporting biomarker that improves the diagnostic accuracy when combined with clinical information, but not when used alone [28]. Also, EEG mismatch negativity (MMN) is a promising tool for monitoring biomarkers to track physiological alterations following interventions and disease progression [29]. Future studies concentrating on the clinical values of microstate dynamics as supporting—but not diagnostic—biomarkers might also be important for the clinical validation of EEG biomarkers.

Overall, the present findings suggest that temporal parameters of microstates B and D in the four-class solution and microstates C and G in the seven-class solution are promising candidates as biomarkers for distinguishing patients with dementia from healthy individuals. While they may not differentiate AD from FTD, they provide a valuable step toward scalable and early screening methods. Future work should aim to expand on these findings by using larger cohorts, prospective designs, and classification analyses to further establish the diagnostic utility and accuracy of microstate-based biomarkers.

## 5. Conclusions

This study shows that different EEG microstate clustering solutions reveal distinct temporal features that distinguish patients with dementia from healthy controls. In particular, the four-class (B, D) and seven-class (C, G) solutions each captured successful aspects of group differentiation, and normalization of temporal parameters further enhanced sensitivity to disease-related changes. Although microstate metrics did not discriminate Alzheimer’s disease from frontotemporal dementia, they reliably distinguished both groups from controls and correlated with cognitive performance.

These findings support EEG microstate analysis as a scalable and noninvasive approach for dementia screening. With the standardized procedure applied in the current study, beyond early detection, microstate-derived metrics could help track disease progression or treatment response in longitudinal studies. Future work should validate these results in larger, multicenter datasets; evaluate diagnostic performance parameters such as sensitivity and specificity using receiver operating characteristic curves; and explore integration with structural and molecular biomarkers to advance personalized diagnostic frameworks in neurodegenerative disorders.

## Figures and Tables

**Table 1:** Shared variance between the identified microstate maps in the current dataset and published microstate maps in 4-class clustering solution.

	A (GrandMean)	B (GrandMean)	C (GrandMean)	D (GrandMean)
A (MetaMaps)	97.35	0.33	34.02	50.09
B (MetaMaps)	3.08	98.70	39.77	41.17
C (MetaMaps)	33.95	39.61	96.86	67.93
D (MetaMaps)	20.86	29.12	23.67	86.54

**Table 2:** Group differences in temporal parameter of 4-class microstates.

	F	*p*	Nonparametric *p*
Duration_A	2.391	2.156	1.342
**Duration_B**	8.887	0.022	0.022
Duration_C	4.116	0.44	0.308
Duration_D	1.529	4.906	1.408
DurationAll	2.175	2.64	1.386
Occurrence_A	1.548	4.818	4.51
Occurrence_B	6.638	0.044	0.088
Occurrence_C	1.229	6.556	2.596
**Occurrence_D**	10.199	0.022	0.022
OccurrenceAll	2.575	1.804	1.386
Coverage_A	0.818	9.79	4.752
**Coverage_B**	13.241	0.022	0.022
Coverage_C	1.758	3.938	5.434
Coverage_D	5.991	0.088	0.022
Relative Duration_A	0.813	9.834	3.762
**Relative Duration_B**	8.685	0.022	0.022
Relative Duration_C	1.424	5.434	8.91
**Relative Duration_D**	6.67	0.044	0.022
Relative Occurrence_A	0.343	15.62	5.522
**Relative Occurrence_B**	15.93	0.022	0.022
Relative Occurrence_C	2.687	1.628	1.364
Relative Occurrence_D	6.096	0.066	0.022

Age and gender were included in the ANCOVA model as covariates. Significant parameters are marked in bold.

**Table 3:** Linear regression on MMSE scores with each temporal parameter of 4-class microstates.

	Unstandardized Coefficient	SE	t	*p*
Duration B	−193.894	55.828	−3.473	<0.001
Occurrence D	3.377	0.826	4.089	<0.001
Coverage B	−0.287	0.097	−2.971	0.004
Relative Duration B	−11.348	4.797	−2.366	0.02
Relative Duration D	11.928	3.307	3.607	<0.001
Relative Occurrence B	−50.812	15.643	−3.248	0.002

Age and gender were included in the model as covariates. Lower MMSE score represents greater cognitive impairment.

**Table 4:** Shared variance between the identified microstate maps in the current dataset and published microstate maps in 7-class clustering solutions.

	A (Grand Mean)	B (Grand Mean)	C (Grand Mean)	D (Grand Mean)	E (Grand Mean)	F (Grand Mean)	G (Grand Mean)
A (MetaMaps)	99.06	10.27	60.74	51.18	12.87	29.00	19.95
B (MetaMaps)	0.15	87.99	34.77	27.52	32.79	37.46	69.09
C (MetaMaps)	52.05	70.50	95.63	49.42	71.45	1.99	11.68
D (MetaMaps)	33.88	57.56	51.57	95.85	2.62	14.02	0.45
E (MetaMaps)	29.29	44.43	67.46	12.13	92.74	9.79	19.68
F (MetaMaps)	24.15	20.76	0.98	5.48	4.31	97.81	34.02
G (MetaMaps)	27.42	20.35	0.09	6.23	25.04	21.80	92.17

**Table 5:** Group differences in temporal parameter of 7-class microstates.

	F	*p*	Nonparametric *p*
Duration_A	1.987	5.328	2.479
Duration_B	1.877	5.92	2.664
Duration_C	2.765	2.553	0.888
Duration_D	1.059	12.987	12.987
Duration_E	6.56	0.074	0.037
Duration_F	5.879	0.148	0.074
**Duration_G**	9.772	0.037	0.037
DurationAll	1.96	5.439	1.332
Occurrence_A	2.198	4.366	3.885
Occurrence_B	1.495	8.51	9.361
**Occurrence_C**	17.085	0.037	0.037
Occurrence_D	5.252	0.259	0.148
Occurrence_E	0.897	15.244	12.95
Occurrence_F	4.102	0.74	0.518
Occurrence_G	6.495	0.074	0.111
OccurrenceAll	2.44	3.478	1.332
Coverage_A	0.623	19.943	19.98
Coverage_B	0.863	15.762	35.224
**Coverage_C**	9.33	0.037	0.037
Coverage_D	1.809	6.29	1.221
Coverage_E	3.053	1.961	1.11
Coverage_F	5.948	0.148	0.037
**Coverage_G**	10.012	0.037	0.037
Relative Duration_A	0.037	35.668	35.594
Relative Duration_B	2.437	3.478	11.544
**Relative Duration_C**	10.927	0.037	0.037
Relative Duration_D	0.232	29.341	11.063
Relative Duration_E	2.296	3.959	1.036
Relative Duration_F	3.908	0.888	0.037
**Relative Duration_G**	7.867	0.037	0.037
Relative Occurrence_A	1.203	11.322	10.767
Relative Occurrence_B	0.22	29.711	31.598
**Relative Occurrence_C**	9.483	0.037	0.037
Relative Occurrence_D	2.918	2.22	0.629
Relative Occurrence_E	2.761	2.553	2.22
Relative Occurrence_F	5.542	0.222	0.037
**Relative Occurrence_G**	10.226	0.037	0.037

Age and gender were included in the ANCOVA model as covariates. Significant parameters are marked in bold.

**Table 6:** Linear regression on MMSE scores with each temporal parameter of 7-class microstates.

	Unstandardized Coefficient	SE	t	*p*
Duration_G	−227.462	63.105	−3.605	<0.001
Occurrence_C	2.784	0.79	3.526	<0.001
Coverage_C	0.178	0.078	2.281	0.025
Coverage_G	−0.323	0.123	−2.622	0.01
Relative Duration_C	11.876	5.014	2.369	0.02
Relative Duration_G	−13.392	4.995	−2.681	0.009
Relative Occurrence_C	26.952	11.976	2.251	0.027
Relative Occurrence_G	−40.831	15.928	−2.563	0.012

Age and gender were included in the model as covariates.

## Data Availability

The original dataset can be found in OpenNeuro: https://doi.org/10.18112/openneuro.ds004504.v1.0.8.

## Abbreviations

The following abbreviations are used in this manuscript:

EEG	Electroencephalography
AD	Alzheimer’s disease
FTD	Frontotemporal dementia
RSN	Resting-state network

## References

[1] Knopman D.S., Amieva H., Petersen R.C., Chételat G., Holtzman D.M., Hyman B.T., Nixon R.A., Jones D.T. (2021). Alzheimer disease. Nat. Rev. Dis. Primers.

[2] Woolley J.D., Khan B.K., Murthy N.K., Miller B.L., Rankin K.P. (2011). The diagnostic challenge of psychiatric symptoms in neurodegenerative disease: Rates of and risk factors for prior psychiatric diagnosis in patients with early neurodegenerative disease. J. Clin. Psychiatry.

[3] Erkkinen M.G., Kim M.O., Geschwind M.D. (2018). Clinical neurology and epidemiology of the major neurodegenerative diseases. Cold Spring Harb. Perspect. Biol..

[4] Rabinovici G.D., Seeley W.W., Kim E.J., Gorno-Tempini M.L., Rascovsky K., Pagliaro T.A., Allison S.C., Halabi C., Kramer J.H., Johnson J.K. (2007). Distinct MRI atrophy patterns in autopsy-proven Alzheimer’s disease and frontotemporal lobar degeneration. Am. J. Alzheimer’s Dis. Other Dement..

[5] Gudmundsson S., Runarsson T.P., Sigurdsson S., Eiriksdottir G., Johnsen K. (2007). Reliability of quantitative EEG features. Clin. Neurophysiol..

[6] Gyurkovics M., Clements G.M., Low K.A., Fabiani M., Gratton G. (2021). The impact of 1/f activity and baseline correction on the results and interpretation of time-frequency analyses of EEG/MEG data: A cautionary tale. NeuroImage.

[7] Michel C.M., Koenig T. (2018). EEG microstates as a tool for studying the temporal dynamics of whole-brain neuronal networks: A review. NeuroImage.

[8] Lehmann D., Ozaki H., Pal I. (1987). EEG alpha map series: Brain micro-states by space-oriented adaptive segmentation. Electroencephalogr. Clin. Neurophysiol..

[9] Michel C.M., Brechet L., Schiller B., Koenig T. (2024). Current state of EEG/ERP microstate research. Brain Topogr..

[10] Kleinert T., Koenig T., Nash K., Wascher E. (2024). On the reliability of the EEG microstate approach. Brain Topogr..

[11] Nishida K., Morishima Y., Yoshimura M., Isotani T., Irisawa S., Jann K., Dierks T., Strik W., Kinoshita T., Koenig T. (2013). EEG microstates associated with salience and frontoparietal networks in frontotemporal dementia, schizophrenia and Alzheimer’s disease. Clin. Neurophysiol..

[12] Lin N., Gao J., Mao C., Sun H., Lu Q., Cui L. (2021). Differences in multimodal electroencephalogram and clinical correlations between early-onset Alzheimer’s disease and frontotemporal dementia. Front. Neurosci..

[13] Grieder M., Koenig T., Kinoshita T., Utsunomiya K., Wahlund L.O., Dierks T., Nishida K. (2016). Discovering EEG resting state alterations of semantic dementia. Clin. Neurophysiol..

[14] Khanna A., Pascual-Leone A., Farzan F. (2014). Reliability of resting-state microstate features in electroencephalography. PLoS ONE.

[15] Koenig T., Diezig S., Kalburgi S.N., Antonova E., Artoni F., Brechet L., Britz J., Croce P., Custo A., Damborská A. (2024). EEG-Meta-Microstates: Towards a more objective use of resting-state EEG microstate findings across studies. Brain Topogr..

[16] Nagabhushan Kalburgi S., Kleinert T., Aryan D., Nash K., Schiller B., Koenig T. (2024). MICROSTATELAB: The EEGLAB toolbox for resting-state microstate analysis. Brain Topogr..

[17] Miltiadous A., Tzimourta K.D., Afrantou T., Ioannidis P., Grigoriadis N., Tsalikakis D.G., Angelidis P., Tsipouras M.G., Glavas E., Giannakeas N. (2023). A dataset of scalp EEG recordings of Alzheimer’s disease, frontotemporal dementia and healthy subjects from routine EEG. Data.

[18] Kurlowicz L., Wallace M. (1999). The Mini-Mental State Examination (MMSE). J. Gerontol. Nurs..

[19] Bell C.C. (1994). DSM-IV: Diagnostic and statistical manual of mental disorders. JAMA.

[20] McKhann G., Drachman D., Folstein M., Katzman R., Price D., Stadlan E.M. (1984). Clinical diagnosis of Alzheimer’s disease: Report of the NINCDS-ADRDA Work Group under the auspices of Department of Health and Human Services Task Force on Alzheimer’s Disease. Neurology.

[21] Delorme A., Makeig S. (2004). EEGLAB: An open source toolbox for analysis of single-trial EEG dynamics including independent component analysis. J. Neurosci. Methods.

[22] Britz J., Van De Ville D., Michel C.M. (2010). BOLD correlates of EEG topography reveal rapid resting-state network dynamics. NeuroImage.

[23] Khanna A., Pascual-Leone A., Michel C.M., Farzan F. (2015). Microstates in resting-state EEG: Current status and future directions. Neurosci. Biobehav. Rev..

[24] Tarailis P., Koenig T., Michel C.M., Griškova-Bulanova I. (2024). The Functional Aspects of Resting EEG Microstates: A Systematic Review. Brain Topogr..

[25] Van de Ville D., Britz J., Michel C.M. (2010). EEG microstate sequences in healthy humans at rest reveal scale-free dynamics. Proc. Natl. Acad. Sci. USA.

[26] Musso F., Brinkmeyer J., Mobascher A., Warbrick T., Winterer G. (2010). Spontaneous brain activity and EEG microstates: A novel EEG/fMRI analysis approach to explore resting-state networks. NeuroImage.

[27] Babiloni C., Arakaki X., Azami H., Bennys K., Blinowska K., Bonanni L., Bujan A., Carrillo M.C., Cichocki A., de Frutos-Lucas J. (2021). Measures of resting state EEG rhythms for clinical trials in Alzheimer’s disease: Recommendations of an expert panel. Alzheimer’s Dement. J. Alzheimer’s Assoc..

[28] Stein M.A., Snyder S.M., Rugino T.A., Hornig M. (2016). Commentary: Objective aids for the assessment of ADHD—Further clarification of what FDA approval for marketing means and why NEBA might help clinicians. A response to Arns et al. (2016). J. Child. Psychol. Psychiatry.

[29] Todd J., Salisbury D., Michie P.T. (2023). Why mismatch negativity continues to hold potential in probing altered brain function in schizophrenia. PCN Rep..

